# Constipation, the sole presentation of primary renal carcinoid tumor: A case report

**DOI:** 10.1177/2036361319878915

**Published:** 2019-10-23

**Authors:** Mehdi Salehipour, Amir Ahmad Mostaghni, Bita Geramizadeh, Alireza Makarem, Alireza Rezvani

**Affiliations:** 1 Department of Urology, Shiraz University of Medical Sciences, Shiraz, Iran; 2 Department of Internal Medicine, Shiraz University of Medical Sciences, Shiraz, Iran; 3 Department of Pathology, Transplant Research Center, Shiraz University of Medical Sciences, Shiraz, Iran

**Keywords:** Carcinoid tumor, renal mass, renal carcinoid tumor, constipation

## Abstract

Primary renal carcinoid tumors are quite rare. The pathogenesis of these tumors is unknown due to lack of enterochromaffin cells in the kidney. Because of nonspecific clinical manifestations and radiologic features, they are commonly misdiagnosed. Hence, Primary renal carcinoid tumors should be considered in differential diagnosis of any renal mass. In the present case, a 26-year-old woman was presented with a renal mass and constipation. After partial nephrectomy, diagnosis of carcinoid tumor was confirmed.

## Introduction

Carcinoid tumors are neuroendocrine tumors that arise from the enterochromaffin cells.^1^ Primary renal carcinoid tumors (PRCTs) are externally rare because the enterochromaffin cells are absent in the renal parenchyma.

The clinical manifestations of PRCTs are usually nonspecific, and sometimes they are found incidentally on radiologic studies. We herein report a case of PRCT in a young female who was presented with constipation as the sole symptom of the tumor.

## Case presentation

A 26-year-old woman was presented with a history of constipation for 2 months. The patient had negative history of fever, flashing, diarrhea, sweating, gross hematuria, and any other urinary tract symptoms. Her post-medical history was unremarkable. Physical examination revealed mild abdominal distension.

The laboratory findings were as follows:

Hemoglobin (Hb) = 13 g/dL, Hematocrit (HCT) = 42.3%, white blood cell (WBC) = 9.26 × 10^3^/µL (Neutrophil = 76%, Lymphocyte = 22%, Eosinophil = 3%, Monocyte = 4%, Basophils = 1%), Platelets = 351 × 10^3^/mL, blood urea nitrogen (BUN) = 13 mg/dL, Creatinine = 08 mg/dL, Na = 143 mEq/L, K = 4.9 mEq/L, lactate dehydrogenase (LDH) = 154 Iu/L, and Ferritin = 25.7 ng/mL.

**Figure 1.**
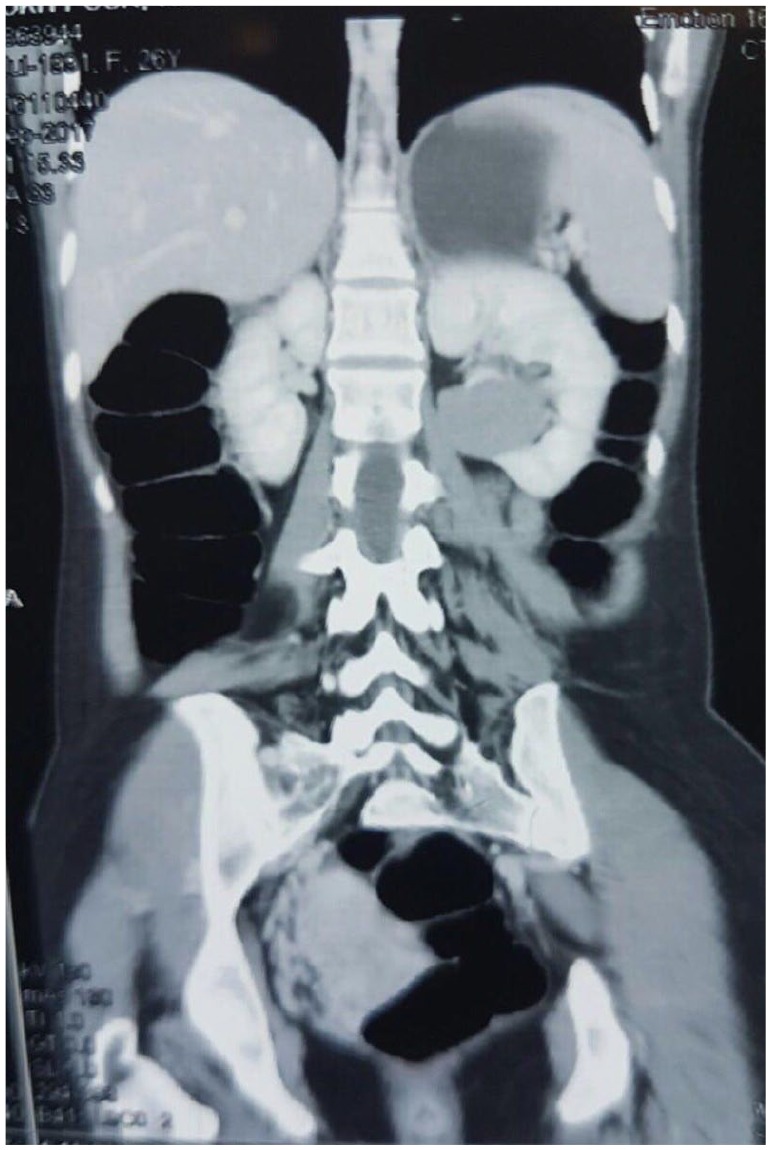
Abdominopelvic spiral CT scan: a 38 mm × 30 mm × 43 mm hypodense solid mass was found in midpole of the left kidney that enhanced after administration of contrast material.

Liver function tests and urinalysis were normal. Chest X-ray was normal, too. Abdominopelvic ultrasonography showed a 30 mm × 33 mm echogenic mass in middle pole of the left kidney. Abdominopelvic spiral CT scan demonstrated a 38 mm × 30 mm × 43 mm hypodense solid mass in the midpole of the left kidney that enhanced after administration of contrast material (Figure 1). Thus, with a preliminary diagnosis of renal malignancy, she underwent left partial nephrectomy through left flunk incision.

**Figure 2.**
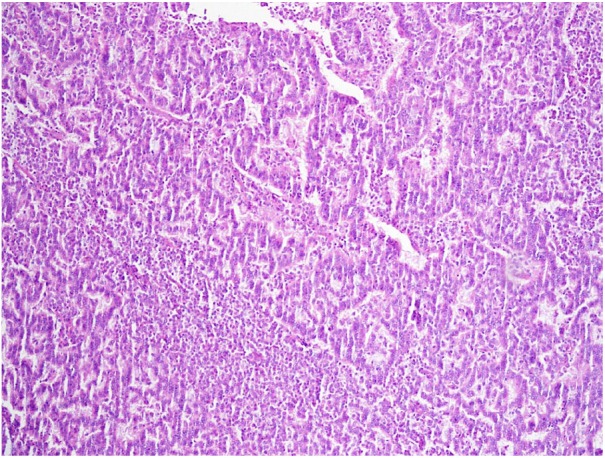
Sections from renal mass show hyper cellular tumor with trabecular and nesting pattern. Individual tumor cells show salt and pepper nuclei (H&EX 250).

**Figure 3.**
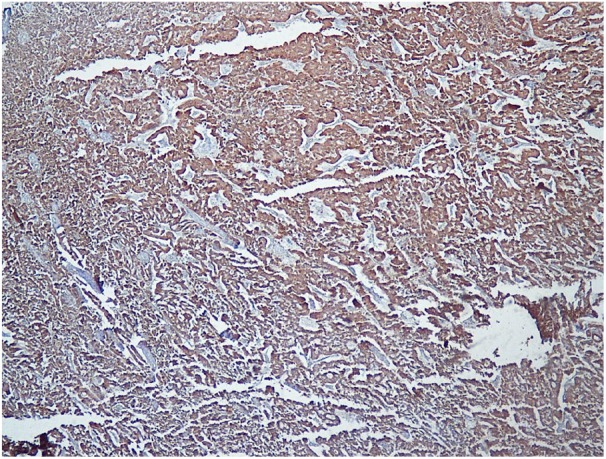
IHC shows positive chromogranin (diffuse and strong).

**Figure 4.**
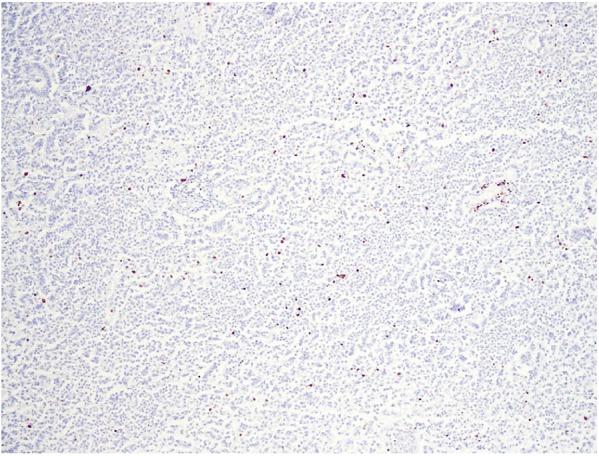
IHC for Ki67 is low (<3%).

Histo-pathologic examination indicated monomorphic cells with trabecular and nesting pattern (Figure 2). Individual cells showed indistinct cytoplasmic borders and nuclei with salt and pepper stippled chromatin. No mitosis or necrosis was seen. Immunohistochemistry was positive for chromogranin and synaptophysin and negative for other renal cell carcinoma (RCC) markers (Figure 3). Proliferative index by Ki67 was low (Figure 4).

After diagnosis of carcinoid tumor, octreotide scan was performed that showed two focal zones of increased radiotracer uptakes in the fourth and eighth segments of the liver. The patient’s chromogranin A was normal, so decision was made for radiofrequency (RF) ablation of the liver lesions. At the 6-month follow-up, the patient remained well.

## Discussion

Carcinoid tumors are low-malignant potential neuroendocrine tumors arising from the enterochromaffin cells.^1^ Carcinoid tumors occur in various organs of the body, with the gastrointestinal tract being the most common site which accounts for 73%, while the respiratory tract accounts for 25% of them.^1^ The testis and prostate are the most common sites of carcinoid tumors in the genitourinary tract.^1,2^ PRCT is a very rare but a well-defined entity. Resnick et al.^3^ reported the first PRCT in 1966. The pathogenesis of PRCT is uncertain because the neuroendocrine cells are not found in the renal parencyme. Several theories regarding the pathogenesis of PRCT have been postulated including:

1. Intestinal metaplasia of the pyelocalyceal urothelium due to chronic inflammation;^1,4^
2. Metastases from unknown primary origin to the kidney;^2,4^
3. Intrapped neural crest or pancreatic cells;^5^
4. Primitive stem cell differentiation.^1,4,6^

Carcinoid tumors of the kidney have been reported in some congenital renal anomalies, among which the most common anomaly is horseshoe kidneys.^6^ Romero et al.^2^ showed that 17.8% of renal carcinoid tumors were associated with horseshoe kidneys, 14.3% with teratomas, and 18% with polycystic kidney disease. In another study, 25% of renal carcinoid tumors were associated with the horseshoe kidney.^1^ The relative risk of carcinoid tumors in individuals with horseshoe kidneys has been calculated between 62 and 120.^7^ Increased occurrence of renal carcinoids in the horseshoe kidney might be related to the existence of aberrant epithelium or teratomatous elements in those kidneys.^1,4^ Hansel et al.^8^ reported that PRCTs were mainly seen in patients younger than 50 years old and there was no tendency in gender and location. On the contrary, Omiyale and Venyo^1^ showed that carcinoid tumors associated with the horseshoe kidneys tend to have a male predilection which could be due to the higher incidence of the horseshoe kidneys in men.

The clinical presentations of PRCTs are often nonspecific and can divided into either localized symptoms such as hematuria and flank pain, or systemic symptoms such as fever, flashing, diarrhea, weight loss, perspiration and constipation.^1,3^ Systemic symptoms could be explained to be due to production and secretion of several hormones. For example, constipation might be due to the secretion of peptide YY.^9^ On the contrary, 25–30% of renal carcinoids were diagnosed as an incidental finding during radiologic investigations. Imaging study of choice for evaluation of the renal masses including renal carcinoid tumors is the helical CT scan. PRCTs show variable and nonspecific findings in the helical CT scan. They may be hyperdense, hypodense, or isodense in comparison with normal renal parenchyma.^4,5^ After administration of contrast material, PRCT may be demonstrated marked, minimal, or no enhancement.^1,5^ Punctate calcification is repeated in some patients.^1^ Somatostatin receptor scintigraphy (SRS) is a useful imaging modality for staging and detecting the recurrence or metastasis of carcinoid tumors.

Radioactive octreotide is a synthetic somatostatin analogue that binds the somatostatin receptors (SR) with high affinity. Although octreotide scintigraphy has a highly reported sensitivity in detecting carcinoid tumors of gastrointestinal origin, its role in PRCTs is not well established since little is known about the prevalence of SR in renal carcinoid tumors. On the contrary, normal renal parenchyma can uptake the trace material and so obscure a suspicious lesion.^5,6,10^ Thus, SRS is not routinely used in pre-operative evaluation of PRCTs.

Complete surgical excision by radical or partial nephrectomy in association with lymphadenectomy is the treatment of choice of PRCTs.^1,5,6^ Whenever the diagnosis of PRCTs is made, metastatic work-up for hidden tumors in other sites must be done. SRS is an important and useful tool in the evaluation of metastatic carcinoid tumors.

Given the paucity of cases, the role of chemotherapy, somatostatin analogues, and targeted therapy with tyrosine kinase inhibitors has not been well established in treatment of metastatic renal carcinoid tumors.^1^ The prognosis of PRCTs seems to be good. The most important prognostic factor is tumor stage at presentation. Tumors confined to the kidney, with size less than 4 cm and age younger than 40 were found to have a good prognosis. PRCTs arising within a horseshoe kidney tend to have less aggressive behavior compared with primary carcinoid tumors originating from a normal kidney.

In conclusion, PRCTs are very rare and should be considered in differential diagnosis of other renal masses. Surgical resection is currently the only treatment. There is limited data about the role of other therapeutic modalities such as chemotherapy and targeted therapy in the treatment of PRCTs. After diagnosis of PRCTs, metastatic work up must be performed to find occult metastases in other organs. Close and long-term follow-up is essential because the indolent course of the disease.
